# Natural-Based Hydrogels for Bone Regeneration: Emerging Technologies and Translational Perspectives

**DOI:** 10.3390/jfb17100498

**Published:** 2026-10-02

**Authors:** Houmam Anees, Paul Maria Merkel, Lukas Boennen, Fahed Al-Arbid, Anas Miqdad, Aria Balouchkhosravi, Ibrahim El Mourabit, Gero Knapp, Markus Rupp, Christian Heiss

**Affiliations:** 1 Department of Trauma, Hand and Reconstructive Surgery, Faculty of Medicine, Justus-Liebig-University of Giessen, 35392 Giessen, Germany; 2 Experimental Trauma Surgery, Faculty of Medicine, Justus-Liebig-University of Giessen, 35392 Giessen, Germany; 3 Department of Orthopaedics and Traumatology, Faculty of Medicine, Biruni University, 34015 Istanbul, Türkiye

**Keywords:** natural-based hydrogels, bone regeneration, bone tissue engineering, regenerative medicine, osteogenesis, angiogenesis, osteoimmunology, 3D bioprinting, clinical translation

## Abstract

Bone defects caused by trauma, infection, tumour resection and impaired healing remain difficult to reconstruct because successful repair requires coordinated mechanical stability, vascularisation, immune regulation and osteogenesis. Natural-based hydrogels provide hydrated, extracellular-matrix-mimicking platforms that can conform to irregular defects and integrate local delivery of bioactive factors, cells, extracellular vesicles and mineral phases. This critical narrative review examines natural-based hydrogels for bone regeneration from a biomaterials-to-clinic perspective, focusing on material design, cell–material interactions, vascularisation, osteoimmunomodulation, controlled therapeutic delivery, injectable and stimuli-responsive systems, composite reinforcement and 3D bioprinting. Human evidence exists for selected gelatin- and hyaluronan-based systems and for clinically adjacent applications such as growth-factor-assisted fracture healing and antibacterial implant coatings; however, most multifunctional regenerative systems remain preclinical. We therefore emphasise translational maturity rather than technological novelty alone. Key barriers include insufficient load-bearing mechanics, variable in vivo degradation, vascularisation at clinically relevant scale, manufacturing and sterilisation constraints, regulatory complexity and limited comparative clinical evidence. The most credible path to clinical adoption is likely to involve indication-specific systems that provide a reproducible biological advantage while remaining surgically compatible, scalable and practically manufacturable.

## 1. Introduction

### 1.1. Clinical Need and Limitations of Current Bone-Reconstruction Strategies

Bone can regenerate without scar formation, but this capacity is finite. High-energy trauma, tumour resection, infection and biologically compromised fracture environments may produce defects that exceed endogenous repair capacity and require reconstructive intervention [1,2]. Delayed union and non-union remain clinically important despite modern fixation; a large epidemiological analysis reported non-union in approximately 5% of fractures, with marked variations in anatomical site, injury severity and patient-related factors [3].

The reconstructive problem is rarely purely biological or purely mechanical. Stable fixation, viable soft tissues, adequate perfusion and a permissive cellular microenvironment are interdependent requirements for healing [4,5]. Autologous bone graft remains the reference biological graft because it combines osteogenic cells, osteoinductive mediators and an osteoconductive matrix, but graft volume is finite and harvest adds morbidity and operative exposure. Allografts provide an osteoconductive structural matrix and, depending on processing and graft type, may offer greater initial structural support than many resorbable substitutes, but they contain few or no viable osteogenic cells and remodel more slowly than autograft. Synthetic substitutes include calcium–phosphate ceramics such as hydroxyapatite and beta-tricalcium phosphate, calcium–sulphate products and bioactive glasses; these are principally osteoconductive and differ substantially in brittleness, resorption rate and capacity to be replaced by new bone [6,7,8].

Hydrogels are three-dimensional, water-swollen networks of hydrophilic polymers held together by physical and/or chemical crosslinks. Their high water content permits molecular transport and can provide an extracellular-matrix-like microenvironment [9,10]. For this review, the term natural-based hydrogel denotes a system in which the principal hydrogel-forming polymeric network is derived from a biological or naturally occurring macromolecule, including collagen, gelatin, fibrin, silk fibroin, hyaluronic acid, alginate, chitosan or tissue-derived extracellular matrix. Chemical functionalisation of a natural polymer (for example, gelatin methacryloyl (GelMA)), use of a synthetic crosslinker, or incorporation of an inorganic or synthetic reinforcing phase does not by itself exclude a formulation from this category; such systems are described explicitly as chemically modified, hybrid or composite natural-based hydrogels. This definition is intended to distinguish them from hydrogels whose primary network is wholly synthetic while acknowledging that many clinically relevant formulations are hybrid materials [11,12,13].

### 1.2. Aim and Translational Perspective

This review examines natural-based hydrogels for bone regeneration from a biomaterials-to-clinic perspective. Rather than cataloguing every reported formulation, we ask four linked questions: what natural hydrogel platforms are available; how can they reproduce the biological requirements of bone repair; which emerging technologies improve their performance; how close are these systems to clinically meaningful use? Particular emphasis is placed on osteogenesis, vascularisation, osteoimmunology, cell–material interactions, fracture healing, critical-sized defects, infected bone defects, advanced manufacturing and the gap between sophisticated preclinical constructs and reproducible clinical products.

**Figure 1:**
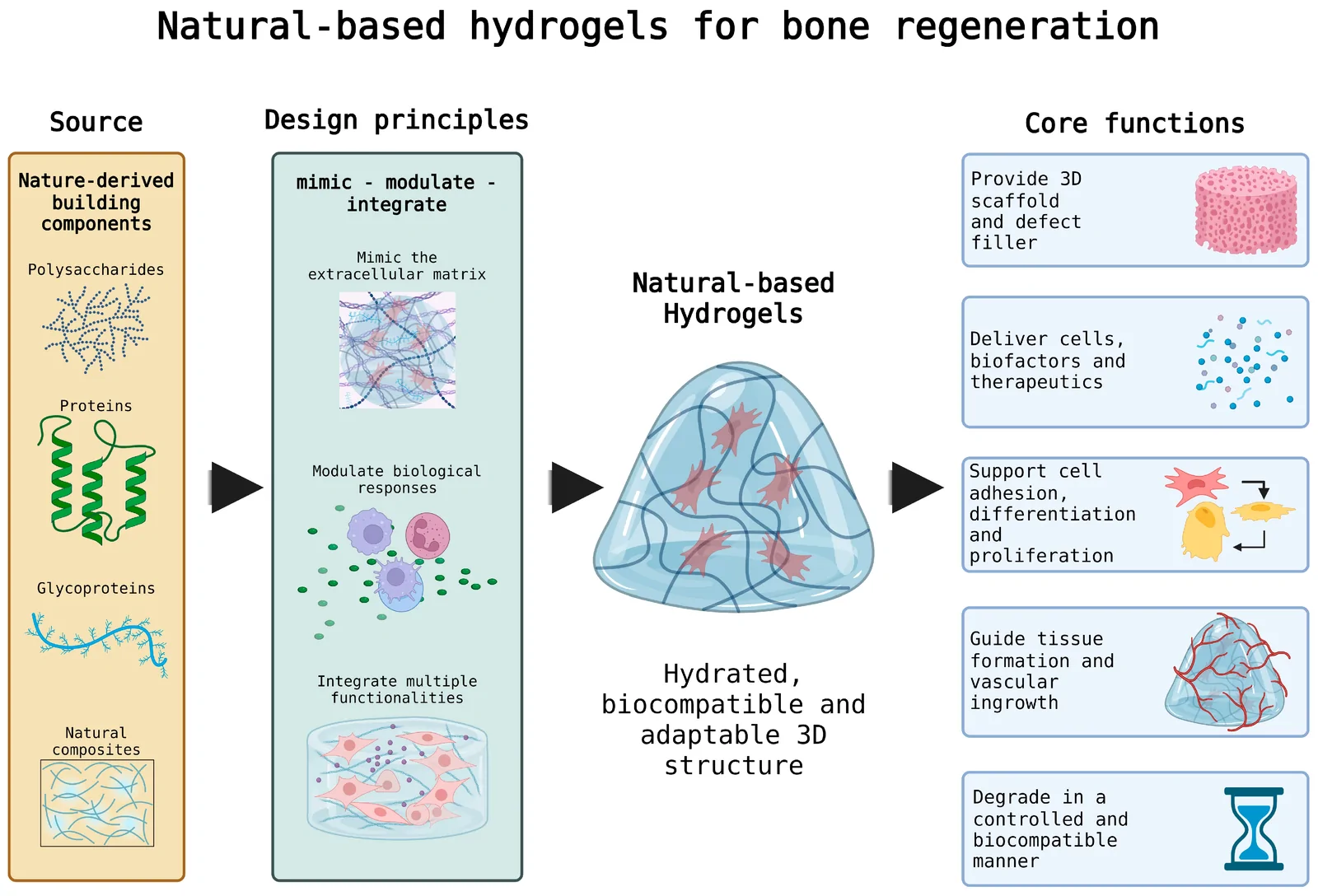
Conceptual framework for natural-based hydrogels in bone regeneration. Natural-derived building components can be engineered to mimic selected extracellular-matrix features, modulate biological responses and integrate multiple functionalities, thereby supporting defect filling, local delivery, cell adhesion and differentiation, vascular ingrowth and controlled degradation. Created with BioRender.com.

The conceptual framework used throughout the review is summarised in Figure 1. Natural-derived building blocks are engineered to mimic selected extracellular-matrix features, modulate biological responses and integrate complementary functions, with the ultimate objective of restoring bone structure and function.

### 1.3. Review Approach

This article was prepared as a critical narrative review rather than a systematic review. Targeted searches were performed iteratively on PubMed/MEDLINE and publisher databases through 15 August 2026. Core combinations included (hydrogel OR natural hydrogel OR natural-based hydrogel) AND (bone regeneration OR bone repair OR bone defect OR fracture healing OR non-union), supplemented by material-specific and translational terms including “gelatin”, “GelMA”, “collagen”, “fibrin”, “silk fibroin”, “hyaluronic acid”, “alginate”, “chitosan”, “decellularised extracellular matrix”, “crosslinking”, “vascularisation”, “osteoimmunology”, “extracellular vesicles”, “infection”, “osteomyelitis”, “bioprinting”, “sterilisation” and “clinical translation”. Backward and forward citation checking of key reviews and primary studies was used to identify additional relevant reports. The eligible literature comprised peer-reviewed English-language original studies and high-quality reviews addressing natural-based hydrogel design, biological mechanisms, bone regeneration or clinically relevant translation. Studies without a substantive hydrogel component or without relevance to bone repair or the translational design questions of this review were not prioritised. For translational statements, particular weight was given to primary human evidence and to large-animal studies addressing clinically relevant defect size or loading. Study selection was qualitative and relevance-based; no formal risk-of-bias tool, PRISMA workflow or systematic-review protocol was used, and the review does not claim exhaustive systematic coverage. High-impact factual claims and reference metadata were re-audited before submission by matching DOI, title and study design to PubMed or publisher records.

## 2. Natural-Based Hydrogel Platforms and Design Principles

### 2.1. Protein-Derived Hydrogels

Protein-derived hydrogels include collagen, gelatin, fibrin and silk fibroin. Their principal advantage is biological familiarity: collagen- and gelatin-based matrices present cell-interactive motifs and can be remodelled enzymatically, while chemical modification allows otherwise soft natural polymers to be crosslinked with much greater control [11]. GelMA is the most widely used example. It retains collagen-derived cell-interactive sequences while methacrylation enables photocrosslinking, tunable stiffness and bioprinting [14]. Photocrosslinking nevertheless requires careful control of wavelength, irradiance, exposure time and photoinitiator concentration. Phototoxicity is not determined by light alone: reactive species generated during polymerisation and residual photoinitiator can reduce cell viability, and lithium phenyl-2,4,6-trimethylbenzoylphosphinate (LAP) exposed to 405 nm light has shown concentration- and exposure-dependent cytotoxicity in mammalian cells [15]. When feasible, longer-wavelength visible-light approaches and the lowest effective light dose may reduce UV-associated damage; however, cytocompatibility must be validated for the complete formulation because initiator chemistry, concentration and exposure remain critical.

Protein-derived systems are nevertheless not intrinsically load bearing. Increasing polymer concentration and effective crosslink density generally improve shape retention and stiffness but reduce swelling and can slow degradation or restrict cell migration. The magnitude is formulation-specific: in one GelMA study, increasing polymer concentration from 5% to 20% increased compressive modulus from 3 ± 1 to 108 ± 8 kPa, while swelling fell from 1476 ± 28% to 470 ± 9% and 56-day degradation decreased from complete degradation to 80 ± 7% [16]. These values should not be treated as universal thresholds because degree of methacrylation, initiator system and light dose also alter network density [14]. Silk-fibroin-based hydrogels offer another route to improve material stability and have supported osteogenic differentiation and bone repair in preclinical models, particularly after functionalisation with bioactive motifs [17]. Their translational role is therefore best understood as an engineerable biological matrix rather than a structural substitute for cortical bone.

### 2.2. Polysaccharide-Derived Hydrogels

Hyaluronic acid (HA), alginate and chitosan are prominent polysaccharide platforms. HA is a native extracellular-matrix glycosaminoglycan with strong water-binding capacity and a substantial literature field in bone tissue engineering; chemical modification is commonly used to improve mechanical stability, control degradation and introduce osteogenic or cell-adhesive functions [18]. Alginate gels readily under mild ionic conditions and is particularly useful for injectable delivery, but unmodified alginate provides limited mammalian cell adhesion. A further limitation of ionically crosslinked alginate is progressive loss of divalent crosslinking ions in physiological media: exchange of Ca2+ with monovalent ions such as Na+ weakens the network and can lead to swelling and relatively rapid in vivo dissolution. Alginate is therefore frequently covalently modified or combined with proteins, peptides, mineral phases or secondary networks when prolonged structural persistence is required [11,19].

Chitosan provides a cationic, biodegradable polysaccharide platform with useful mucoadhesive and antimicrobial characteristics. Native chitosan is poorly soluble near physiological pH because deprotonation of its amino groups reduces aqueous solubility; this constrains direct neutral-pH formulation. Water-soluble derivatives such as carboxymethyl chitosan and glycol chitosan, as well as thermosensitive chitosan-based systems, have therefore been developed to permit preparation and gelation under more physiologically compatible conditions. For example, carboxymethylation combined with alpha-cyclodextrin conjugation produced a water-soluble injectable chitosan derivative capable of gel formation under physiological conditions [20]. Its mechanical limitations and formulation sensitivity have also driven development of injectable chitosan nanocomposites incorporating hydroxyapatite, bioactive glass, metallic or carbon-based phases [21].

### 2.3. Decellularised Extracellular-Matrix-Derived Hydrogels

Decellularised extracellular-matrix (dECM)-derived hydrogels occupy an intermediate position between purified natural polymers and tissue-derived biological scaffolds. After decellularisation, matrix material can be enzymatically solubilised and reconstituted as an injectable hydrogel, retaining a complex mixture of collagenous and non-collagenous matrix components that is difficult to reproduce with a single purified polymer. Bone-derived dECM is particularly attractive because tissue-specific matrix cues may support osteogenic differentiation. Sawkins and colleagues generated hydrogels from demineralised and decellularised bovine bone ECM and demonstrated distinct structural, mechanical and biological properties compared with demineralised bone matrix and collagen controls [22]. In a subsequent study, bone-ECM hydrogels increased osteogenic marker expression in C2C12 myoblasts and primary calvarial cells, including substantial increases in osteopontin and osteocalcin expression [23].

The same biological complexity that makes dECM attractive also creates translational challenges. Tissue source, donor characteristics, decellularisation and demineralisation protocols, residual DNA, retained growth factors, digestion conditions and processing can all alter composition, gelation and biological activity. dECM hydrogels are also generally mechanically weak and are therefore often better considered as bioactive matrices or components of reinforced composites than as independent structural substitutes. For clinical development, compositional release criteria and batch-to-batch reproducibility will be as important as proof of osteogenic activity [22,23].

### 2.4. Crosslinking Chemistry and Translational Implications

Crosslinking strategy determines not only hydrogel mechanics but also cytocompatibility, degradation and manufacturability. Physical and ionic crosslinking can proceed under mild conditions and avoid reactive chemical residues, but network stability may be reversible in physiological fluids, as illustrated by ionically crosslinked alginate. Enzymatic crosslinking can provide cell-compatible reaction conditions and substrate specificity, although enzyme activity, reaction time and residual enzyme become additional process variables. Photocrosslinking offers rapid spatial and temporal control and is highly compatible with bioprinting, but requires validation of photoinitiator concentration, wavelength and light dose because radical generation can injure encapsulated cells [14,15]. Dynamic covalent bonds such as imines or hydrazones combine injectability and self-healing with adaptable degradation, but introduce additional chemistry whose stability and degradation products require characterisation [24,25]. From a translational perspective, the preferred crosslinking route is therefore the least complex chemistry that reproducibly delivers the required handling, mechanics and degradation profile under clinically compatible conditions.

### 2.5. Hybrid and Composite Design as the Dominant Translational Strategy

No single natural polymer simultaneously provides optimal cell interaction, mineral bioactivity, toughness, degradation and printability. Hybridisation is therefore not an exception but increasingly the dominant design strategy. Natural networks are combined with synthetic crosslinkers, interpenetrating networks, calcium–phosphate phases, bioactive glass, nanoclays or other reinforcing components to improve mechanical stability and osteoconduction [12,13]. Within the terminology used here, these remain natural-based only when the natural polymer constitutes the principal hydrogel-forming matrix; the synthetic or inorganic fraction and its functional role should be reported explicitly. The trade-off is that each added component increases the burden of formulation control, sterilisation, toxicology and regulatory characterisation. The principal features and translational roles of these platforms are summarised in Table 1.

## 3. Engineering the Bone-Regenerative Microenvironment

### 3.1. Cell–Material Interactions and Osteogenic Commitment

A hydrogel is biologically active even before a drug or cell is added. Mesenchymal stromal/stem cells (MSCs) sense matrix stiffness, viscoelasticity, topography, ligand density and ligand spacing through integrins, focal adhesions and cytoskeletal tension. These cues interact with FAK, Rho/ROCK, MAPK, YAP/TAZ and Wnt-related signalling networks that influence osteogenic commitment [26,27]. Controlled ligand spacing on soft hydrogels has been shown to restore osteogenic mechanotransduction despite low bulk stiffness, demonstrating that biochemical presentation and mechanics are jointly tunable rather than independent variables [27].

Biochemical osteogenic signalling converges on many of the same transcriptional outputs. BMP receptor signalling through Smad1/5/8 promotes Runx2- and Osterix/Sp7-dependent osteoblast differentiation, with subsequent matrix deposition and mineralisation [28]. For hydrogel design, the implication is straightforward: stiffness, degradability and growth-factor presentation should be optimised together, because an osteoinductive cue cannot compensate for a matrix that prevents cell spreading, migration or tissue replacement.

### 3.2. Vascularisation and Osteoimmunology

Vascularisation is a prerequisite for sustained regeneration in defects that exceed diffusion distances. Hydrogel strategies therefore use VEGF and related angiogenic factors, pro-angiogenic ions, endothelial-cell co-delivery, prevascularisation or channelled architectures to accelerate vessel invasion [29,30]. Osteogenesis and angiogenesis are closely coupled through HIF-1α/VEGF, PDGF, FGF and angiopoietin/Tie signalling, among other pathways [31]. The translational problem is scale: a strategy that vascularises a small cranial construct may not provide timely perfusion through the centre of a large human segmental defect.

Bone healing is also an immune process. Neutrophils, resident osteal macrophages, monocyte-derived macrophages and lymphocyte subsets participate in the inflammatory and reparative sequence of fracture healing [32]. The commonly used M1/M2 terminology is a helpful shorthand but oversimplifies a continuous and context-dependent macrophage phenotype spectrum. Materials can influence this trajectory through surface chemistry, mechanics, degradation products and delivered signals. A glycopeptide hydrogel coating on a PCL/nano-hydroxyapatite scaffold promoted a pro-regenerative macrophage response, improved MSC osteogenic differentiation and enhanced cranial bone repair in vivo [33].

### 3.3. Biofunctionalisation and Controlled Therapeutic Delivery

Biofunctionalisation converts a hydrated matrix into an instructive delivery platform. Growth factors, adhesion ligands, peptides and small molecules can be physically entrapped, covalently immobilised or affinity-sequestered, allowing local presentation at concentrations and time scales that are difficult to achieve by bolus administration [34]. Bone morphogenetic protein-2 (BMP-2) remains a model osteoinductive cargo, but the design problem is not simply whether BMP-2 is present: local retention, release kinetics, cell adhesion and matrix mechanics determine efficacy and safety.

Engineered hydrogels have combined tunable mechanics, RGD-mediated adhesion and controlled BMP-2 release, while HA matrices modified with an integrin-specific fibronectin fragment increased the osteogenic efficacy of delivered BMP-2 [35,36]. Multifactor approaches aim to reproduce angiogenic–osteogenic coupling; a blue-light-curable hydrogel delivering a VEGF-mimetic peptide together with parathyroid hormone (1–34), for example, accelerated bone regeneration in a cranial defect model [37].

The frontier is temporal control. ROS-, pH- and inflammation-responsive networks can alter cargo release as the defect microenvironment changes. Recent systems have used ROS-responsive delivery of dimethyl fumarate and environmentally responsive sequencing of anti-inflammatory and osteogenic signals [38,39]. These designs are biologically appealing, but every additional trigger, drug and linker expands the burden of reproducibility, toxicology and combination-product regulation.

### 3.4. Cells and Extracellular Vesicles

Cell-laden hydrogels provide both a scaffold and a local delivery vehicle. MSCs are extensively studied because they can contribute to osteogenic differentiation and paracrine regulation, yet direct injection is limited by poor retention and survival. Microribbon-based hydrogels improved the spatial microenvironment for transplanted stromal cells and accelerated bone regeneration in a critical-size cranial model [40]. The performance of these constructs depends strongly on matrix degradability, ligand presentation and mechanics, reinforcing the view that cells and scaffold cannot be optimised independently.

Extracellular vesicles (EVs), including exosome-enriched preparations, offer a cell-free route to deliver paracrine signals. Hydrogels can prolong local EV retention; human umbilical cord MSC-derived exosomes incorporated into a hydroxyapatite-containing HA/alginate hydrogel enhanced osteogenic responses and bone regeneration in preclinical models, while black-phosphorus thermosensitive hydrogels have been used for controlled delivery of bone marrow MSC-derived exosomes [41,42]. EV-based systems remain complex products because isolation method, source cell, culture conditions, cargo composition, potency, dose, storage and batch consistency all influence activity [43,44,45].

**Figure 2:**
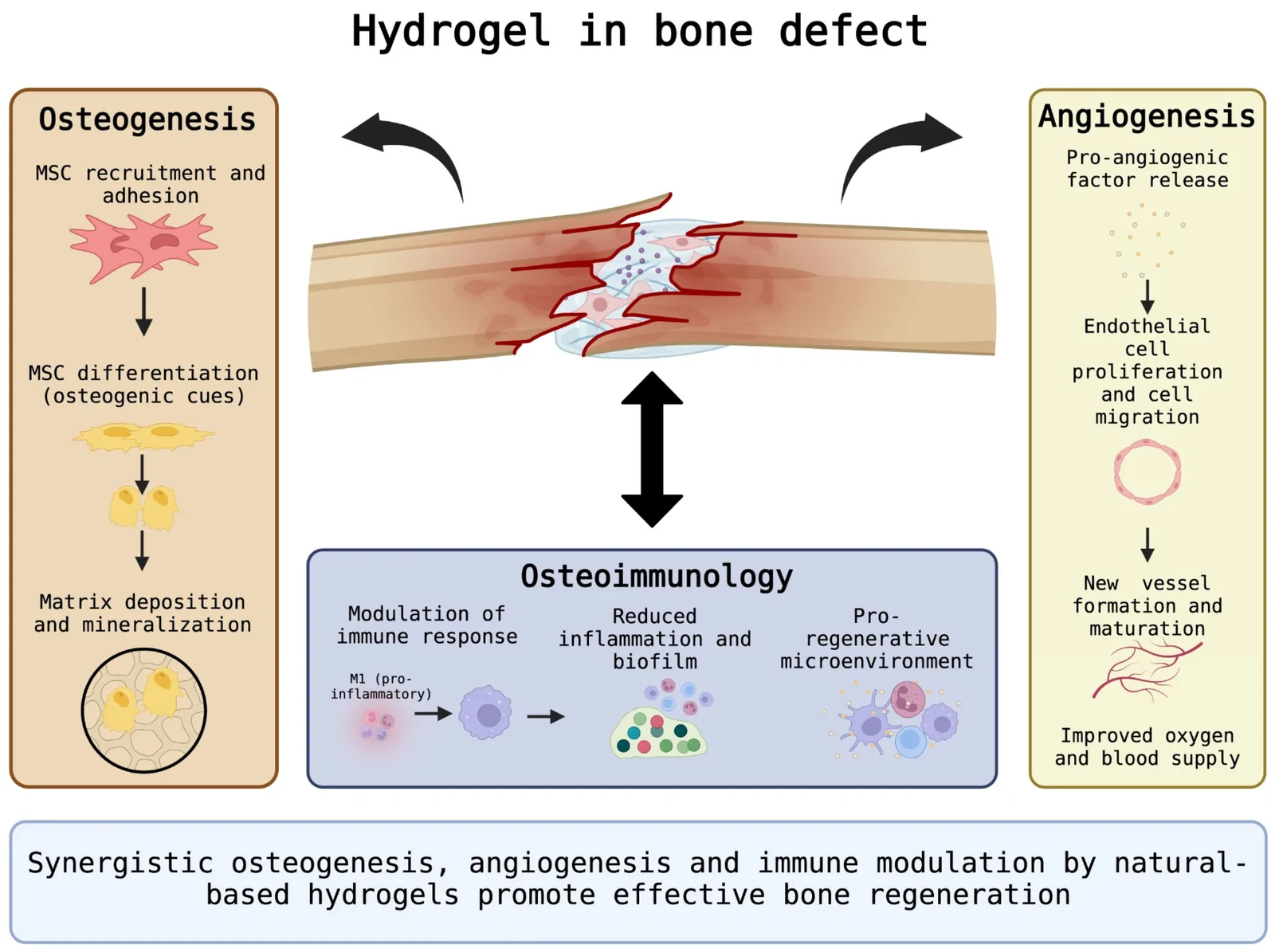
Biological mechanisms relevant to hydrogel-mediated bone regeneration. The schematic summarises osteogenic cell recruitment and differentiation, angiogenic signalling and vascular ingrowth, and immune modulation within the regenerative microenvironment. The M1 label is used as a simplified shorthand within a broader macrophage phenotypic continuum rather than as a strict binary classification. The biofilm-related element is specifically relevant to antimicrobial hydrogel strategies in infected bone environments and should not be interpreted as a universal property of natural-based hydrogels. MSC, mesenchymal stromal/stem cell. Created with BioRender.com.

The interplay of osteogenesis, angiogenesis and osteoimmunology in hydrogel-mediated bone regeneration is summarised in Figure 2.

## 4. Advanced and Emerging Hydrogel Technologies

### 4.1. Injectable, Self-Healing and Stimuli-Responsive Systems

Injectable and self-healing hydrogels are particularly relevant to trauma because they can be delivered through a syringe or cannula, conform to irregular defects and recover network integrity after shear. Dynamic non-covalent interactions and reversible covalent bonds allow a material to shear-thin during delivery and re-form in situ [24]. This can improve surgical handling and retention of encapsulated cells or therapeutics, provided recovery is rapid enough to prevent leakage and gentle enough to preserve biological cargo.

Smart hydrogels respond to endogenous signals such as pH, reactive oxygen species, temperature or enzyme activity, or to exogenous inputs such as light, electric or magnetic fields [46,47,48,49]. Their value lies in converting a changing biological or externally applied stimulus into a controlled change in swelling, degradation, mechanics or therapeutic release. An instructive sequential system recruited endogenous mesenchymal progenitors with platelet-derived growth factor-BB while providing sustained BMP-2; separating the release kinetics improved regeneration while avoiding unfavourable interactions associated with simultaneous presentation [50]. For bone regeneration, the most credible smart systems are those in which responsiveness addresses a defined clinical problem—for example, inflammatory ROS, infection-associated pH or temporally staged factor delivery—without introducing disproportionate manufacturing and regulatory complexity. The important translational question is whether responsive behaviour remains predictable in heterogeneous human wounds rather than only under tightly controlled experimental conditions.

### 4.2. Mineral and Nanocomposite Reinforcement

Composite strategies address the limited mechanics and variable osteoconductivity of pure natural hydrogels by introducing mineral or nanoscale phases. Hydroxyapatite is the most established example because of its chemical similarity to bone minerals; cell-laden nano-hydroxyapatite/protein hydrogels have demonstrated enhanced mineralisation and useful material reinforcement [51]. Bioactive-glass-containing hydrogels can add ion-mediated osteogenic and angiogenic effects, while nanoclays such as Laponite can modify rheology, reinforce the network and release bioactive silicate or magnesium species [52,53].

More experimental particles, including metallic, carbon-based and black-phosphorus materials, introduce conductivity, photothermal responsiveness or controlled phosphate release [13]. Their multifunctionality is appealing, but nanoparticle aggregation or agglomeration can create spatially heterogeneous reinforcement, alter local rheology and printability, produce local concentration peaks and make release or degradation less predictable. Surface chemistry, particle size, ionic strength and mixing sequence therefore require control, and particle dispersion should be characterised rather than assumed from nominal composition. Long-term persistence, degradation products and biosafety must also be considered. Reinforcement should therefore be judged against the intended clinical role: an increase in modulus is valuable if it improves defect retention or mechanobiology, but it does not automatically make an injectable hydrogel a load-bearing implant.

### 4.3. 3D Bioprinting and Patient-Specific Architecture

Three-dimensional printing and bioprinting add spatial control to hydrogel chemistry. Extrusion-, inkjet-, laser- and light-based approaches can create defined pore geometry, channels and regional material composition. Natural polymers including gelatin, alginate, collagen, HA and chitosan are widely used as bioink components and are frequently reinforced with calcium–phosphate or other particulate phases to improve print fidelity and osteoconductivity [54,55].

Patient-specific design is compelling when defect geometry is genuinely complex. Computed-tomography data can be converted into computer-aided design/manufacturing workflows to reproduce craniofacial, tumour-related or traumatic defect geometry [56,57]. Anatomical fidelity, however, does not guarantee biological success: increasing construct size amplifies diffusion limits, vascularisation requirements, manufacturing time and quality-control demands.

Printed architecture is itself cell-instructive. Pore shape, curvature and confinement influence cytoskeletal organisation and mechanotransduction; comparative 3D-printed pore morphologies have altered RhoA/ROCK2 signalling, MSC osteogenic behaviour and subsequent bone formation in vivo [58]. There is no universal optimal pore geometry or dimension for a hydrogel-based bone construct. Larger and more interconnected pores generally facilitate vascular and cellular ingrowth, whereas smaller pores can improve surface area and initial matrix retention; the preferred architecture therefore depends on material stiffness, degradation, cell source, vascularisation strategy, anatomical site and printing resolution [56,58]. Consequently, reported pore dimensions should be interpreted together with geometry, interconnectivity and the mechanical environment rather than as an isolated optimisation target.

## 5. From Experimental Regeneration to Clinical Application

### 5.1. What Has Reached Patients?

The clinical landscape is considerably narrower than the experimental literature. Translation has been most successful when a hydrogel performs a clear, bounded function—local delivery, coating, defect conformity or improvement of graft handling—rather than attempting to replace the complete mechanical and biological functions of bone [10,59]. Human evidence also shows that the blanket statement that hydrogels lack clinical bone-regeneration data is no longer correct.

A prospective randomised cranial study evaluated a hyaluronan-based hydrogel with and without BMP-2 in standardised calvarial defects and reported substantial defect healing without treatment-related local or systemic adverse effects [60]. In fresh tibial shaft fractures treated with intramedullary nailing, a randomised double-blind placebo-controlled trial found higher cumulative radiographic union with local recombinant human fibroblast growth factor-2 (rhFGF-2) delivered in a biodegradable gelatin hydrogel than with hydrogel alone, although time to painless full-weight bearing did not differ significantly [61].

Antibacterial hydrogels provide another clinically mature edge. In a multicentre randomised trial of internal osteosynthesis, a fast-resorbable antibiotic-loaded hyaluronan hydrogel coating reduced post-surgical infection and did not detectably interfere with fracture healing [62]. A separate randomised arthroplasty study also reported fewer early infections with an antibiotic-loaded hydrogel coating [63]. A 2026 network meta-analysis including 26 comparative studies and 3592 patients found Defensive Antibacterial Coating (DAC) hydrogel among coating strategies associated with lower infection risk, although only three included studies were randomised and most evidence remained observational [64].

### 5.2. Fracture Healing, Non-Union and Critical-Sized Defects

Many proof-of-concept hydrogel studies use calvarial or craniofacial models. The term critical-sized defect should not be interpreted as a universal dimensional threshold: criticality is model-specific and depends on species, age, anatomical site, defect geometry, periosteal and soft-tissue preservation, fixation or mechanical stability and the observation period [2,65]. Defects labelled critical-sized across different studies are therefore not necessarily biologically or mechanically equivalent. Calvarial models remain valuable for mechanistic and regenerative proof of concept but do not reproduce fatigue, torsion, fixation-interface behaviour or the mechanical environment of a human tibial or femoral segmental defect. Cell-laden adhesive hydrogels and prevascularised bioprinted constructs have shown strong regenerative effects in craniofacial and femoral animal models, but direct extrapolation to major load-bearing reconstruction remains inappropriate [66,67].

The smaller body of large-animal work is therefore particularly informative. A self-crosslinking calcium–phosphate/hydrogel construct delivering BMP-2 promoted regeneration in a canine critical-size segmental non-union model [68]. Non-human primate work using a gelatin hydrogel carrier for FGF-2 demonstrated accelerated fracture healing and increased mechanical strength, and this translational line later progressed to the randomised human tibial-shaft-fracture trial described above [61,69].

Human long-bone evidence is therefore not absent, but it remains indication-specific. Within the literature identified by our targeted pre-submission search through 15 August 2026, we did not identify a randomised clinical trial in established load-bearing long-bone non-union or a critical-sized post-traumatic segmental defect in which a natural-polymer hydrogel served as the primary regenerative platform. The clinically relevant unresolved question is whether biological advantages demonstrated in acute fractures and preclinical large defects can be reproduced at the scale, mechanics and heterogeneity of major reconstructive trauma.

### 5.3. Fracture-Related Infection, Osteomyelitis and Infected Bone Defects

Infected bone defects add a second objective: infection control must be achieved without creating an environment that prevents reconstruction. Modern fracture-related infection (FRI) principles emphasise surgical debridement, appropriate microbiological sampling, fracture stability, soft-tissue management and systemic antimicrobial therapy tailored to the clinical strategy [70,71]. Local antimicrobial strategies can be valuable adjuncts, particularly for dead-space management and high local drug exposure [72].

Hydrogels are attractive because a single injectable material can occupy dead space, adhere to irregular surfaces and deliver an antimicrobial locally. A thermosensitive chitosan-based hydrogel/nanoparticle system provided sustained vancomycin delivery and improved infection control in an experimental osteomyelitis model [73]. Johnson and colleagues used an injectable hydrogel to deliver lysostaphin; in a murine infected-fracture model, the system eliminated Staphylococcus aureus infection while supporting fracture healing [74].

Recent work has moved beyond antibiotic depots towards simultaneous control of infection, immunity and regeneration. A 2026 injectable hydrogel study reported metabolic reprogramming and protection against reinfection while supporting repair, and temporally immunomodulatory hydrogels have been designed to sequence infection control, immune transition and osteogenesis [75,76]. These are scientifically compelling but high-complexity combination products. Reviews published in 2024–2026 still identify predominantly in vitro and animal evidence for regenerative hydrogel treatment of infected bone [77,78,79,80]. Representative strategies across different evidence levels are summarised in Table 2.

## 6. Why Clinical Translation Remains Limited

### 6.1. Mechanics, Degradation and Biological Scale

Mechanical competence remains the most obvious barrier in long-bone reconstruction. Natural-polymer hydrogels are generally too compliant to act as independent structural substitutes. Double networks, mineral reinforcement and increased crosslink density can improve modulus and toughness but may reduce porosity, cell migration, degradability or injectability [81,82]. The clinically relevant target is not necessarily cortical-bone-equivalent stiffness; it is a predictable function within the mechanical environment created by fixation.

Degradation is equally important. Natural polymers are degraded through hydrolytic and/or enzyme-mediated processes whose rates vary with crosslinking, inflammation, vascularity and tissue composition. A formulation that persists appropriately in a healthy small-animal defect may behave differently in a protease-rich infected bed or a hypovascular non-union. Dynamic covalent strategies can tune network stability, but the added chemistry and degradation products must themselves be characterised [9,25].

Biological scale is a related but distinct problem. Larger defects impose longer diffusion distances, greater vascular demand and more heterogeneous strain fields. Repeated success in permissive small-animal calvarial models therefore cannot substitute for progressive validation in large, loaded and clinically representative defects. The decisive preclinical question is not whether a construct forms bone, but whether it does so under the anatomical, vascular and mechanical constraints relevant to the intended indication.

### 6.2. Manufacturing, Sterilisation and Regulation

Laboratory protocols frequently depend on narrowly controlled polymer batches, bespoke functionalisation and fresh biological cargo. For natural polymers, raw-material identity is itself a critical material attribute. Collagen and gelatin properties vary with species, tissue source, extraction method and Bloom strength; in a direct comparison, GelMA prepared from bovine, porcine and fish gelatin showed markedly different compressive properties, with reported values of approximately 60, 40 and 10 kPa, respectively [83]. HA molecular weight influences viscosity, network formation and cell interactions; alginate behaviour depends strongly on molecular weight and mannuronic/guluronic-acid composition; chitosan solubility, charge density and degradation are affected by molecular weight and degree of deacetylation [11,18,21]. Clinical production therefore requires predefined source specifications and acceptance ranges rather than treating polymer name alone as sufficient material identity.

Sterilisation must likewise be treated as a formulation variable rather than a final packaging step. Steam or other heat-based methods can denature protein-derived polymers and alter network structure; ionising radiation can cause polymer-chain scission or additional crosslinking, thereby changing molecular weight, swelling, rheology, mechanical strength and degradation; ethylene oxide can leave reactive residues and requires effective desorption. UV treatment has limited penetration and can itself alter photosensitive chemistries, whereas sterile filtration is restricted to soluble precursor components that can pass through the membrane. A dedicated review of hydrogel sterilisation concluded that terminal methods can substantially alter functional properties and that the optimal method is material- and formulation-specific [84]. Consequently, sterilisation should be selected early in development and validated against predefined chemical, rheological, mechanical, degradation and biological acceptance criteria.

Regulatory burden rises with biological complexity. An acellular material whose principal mode of action is physical may follow a device pathway, whereas addition of a pharmacologically active growth factor, viable cells or other biological components can trigger combination-product or advanced-therapy requirements depending on jurisdiction and intended mode of action [59,85]. Classification affects the preclinical package, manufacturing standards, pharmacology/toxicology requirements and development cost.

### 6.3. The Complexity–Translatability Paradox

A recurrent pattern across the field can be framed as a complexity–translatability paradox. Adding cells, EVs, nanoparticles, multiple growth factors and responsive chemistries can improve preclinical performance, yet each additional component creates new sources of batch variability, release uncertainty, toxicology, storage burden and regulatory classification [10,59,86]. This is not an argument for simplicity as an end in itself. Rather, every added function should earn its manufacturing and regulatory burden by producing a clinically meaningful improvement that cannot be achieved with a simpler formulation.

This principle also changes how efficacy should be judged. A multifunctional construct that marginally improves micro-computed-tomography bone volume in a rodent defect may be less clinically valuable than a simpler injectable system that can be terminally sterilised, stored and delivered through a standard procedure and that reproducibly improves union or infection outcomes. Translational design should therefore optimise the complete therapeutic pathway, not only the biological endpoint measured in a preclinical model.

### 6.4. Clinical Evidence and Trial Design

Clinical evidence remains the decisive gap for major regenerative indications. The field contains many sophisticated formulations that outperform controls in small animals but few studies compare a hydrogel strategy with contemporary orthopaedic reconstruction under clinically representative conditions [87,88]. Future trials require indication-specific endpoints such as union, time to unrestricted weight bearing, reoperation, infection recurrence, patient-reported outcomes and cost rather than relying predominantly on surrogate imaging measures. Comparators must also be clinically credible, which for many major defects means autograft or an established composite reconstruction rather than an empty defect.

## 7. Future Directions: What Is Most Likely to Reach the Clinic?

### 7.1. Indication-First and Mechanically Integrated Design

A clinically useful next-generation hydrogel should be designed backwards from the indication. For a contained metaphyseal void, injectability and osteoconduction may dominate; for a large segmental defect, fixation integration, vascularisation and controlled space maintenance become central; for fracture-related infection, dead-space management and antimicrobial activity must be integrated with a later regenerative phase. This indication-first approach avoids combining attractive laboratory functions without a clear surgical use case.

Mechanics and biology should therefore be engineered as one therapeutic system. The hydrogel does not need to replace fixation to be clinically valuable; it needs to remain where it is placed, tolerate the local strain environment, deliver the intended biological function and disappear as stable tissue replaces it. This framing favours modular constructs in which established implants provide structural stability while the hydrogel supplies defect-specific biological activity.

### 7.2. Smart, Cell-Free and Osteoimmunomodulatory Systems

Future smart systems are likely to be most translatable when responsiveness replaces rather than simply adds components. Endogenous pH, ROS or enzyme activity can sequence therapeutic release, while cell-free EV or protein-based approaches may reduce some logistical burdens associated with viable-cell products. Recent multifunctional hydrogels demonstrate coordinated angiogenic, osteogenic and immunomodulatory effects, including temporally programmed immune transitions in infected bone [76,89,90,91].

Osteoimmunomodulation will remain important, but the objective should not be permanent suppression of inflammation or a simplistic shift to a single macrophage state. A 2026 review of macrophage-regulating hydrogels emphasised the need to align immune effects with bone-specific mechanics, degradation and spatiotemporal control [92]. Recent systems have coordinated macrophage reprogramming, angiogenesis and osteogenesis in both aseptic and infected models [93,94,95].

### 7.3. Patient-Specific Manufacturing and AI-Assisted Optimisation

Personalised manufacturing is most compelling where defect geometry genuinely affects reconstruction. Imaging-based design and bioprinting can generate constructs that match individual craniofacial, tumour-related or segmental defects [57]. A recent oxygen self-supplying 3D-printed bioactive hydrogel scaffold promoted vascularised bone regeneration without exogenous cells or growth factors, illustrating how material design may simplify the biological payload while retaining function [96].

Artificial intelligence and machine learning can help navigate the multidimensional design space of polymer composition, crosslinking, porosity, mechanics and release kinetics [97]. Machine-learning-assisted inverse design has also been used to generate scaffold architectures with predefined mechanical characteristics and graded structures [98,99]. The limiting factor is data quality: predictive models will only be clinically useful if trained on standardised, reproducible material and biological datasets.

### 7.4. A Translational Roadmap

**Figure 3:**
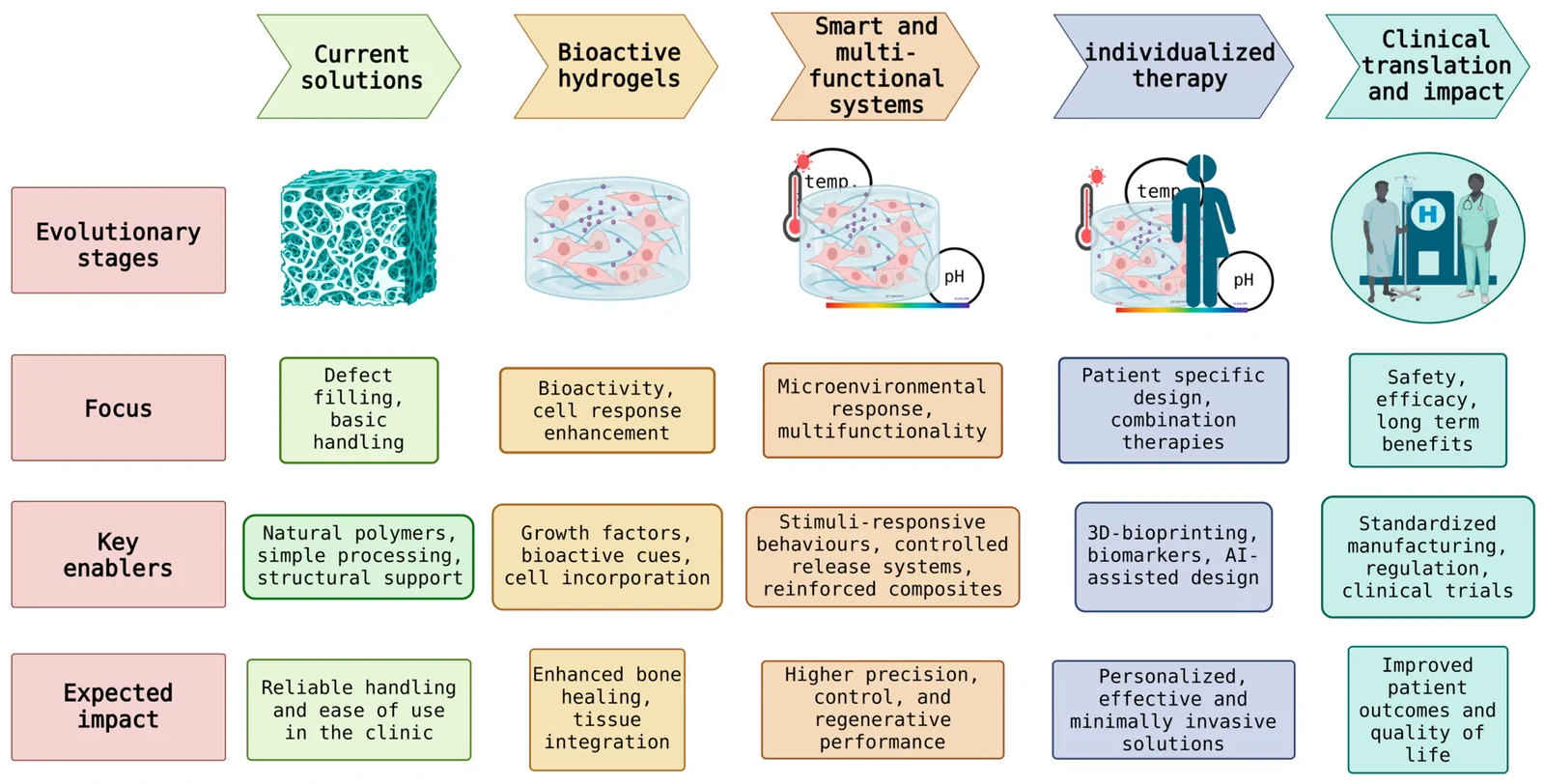
Conceptual translational roadmap from current supportive materials to bioactive, smart, personalised and clinically translated hydrogel systems. The roadmap is illustrative rather than a linear evidence hierarchy: greater functional sophistication may improve biological control but can also increase manufacturing, reproducibility and regulatory requirements. Successful translation therefore depends on demonstrating clinically meaningful benefit while maintaining scalable processing and robust validation. AI, artificial intelligence. Created with BioRender.com.

Three priorities emerge. First, preclinical models should progressively reproduce human defect size, vascular compromise, infection status and loading. Second, manufacturability, sterilisation and regulatory classification should be considered during material design rather than after efficacy has been demonstrated. Third, increasing sophistication should be accompanied by evidence that each added function materially improves a clinically relevant endpoint. Figure 3 illustrates this progression as a conceptual, non-linear roadmap from supportive materials through bioactive, smart and personalised systems towards clinical translation.

## 8. Conclusions

Natural-based hydrogels have evolved from hydrated carriers into bioinstructive platforms capable of integrating local delivery, mineral reinforcement, cellular- or extracellular-vesicle signals, mechanobiological control and additive manufacturing. Their principal strength is not any single polymer or technology, but the ability to create a defect-adapted microenvironment in which osteogenesis, vascularisation and immune regulation can be coordinated. Human studies already demonstrate clinically measurable effects for selected gelatin- and hyaluronan-based systems, while most multifunctional constructs for major load-bearing or infected defects remain preclinical.

The next phase of the field should therefore be judged by translation rather than complexity alone. Clinically representative models, integration with fixation or other established reconstructive strategies, reproducible manufacture, controlled degradation and prospective comparative trials are more likely to determine success than further expansion of the catalogue of possible additives. Natural-based hydrogels are best positioned to enter clinical practice as indication-specific, surgically compatible regenerative components that deliver a clear biological advantage without unnecessary product complexity.

## Figures and Tables

**Table 1:** Natural-based hydrogel platforms relevant to bone regeneration. Translational value depends on the complete formulation rather than polymer origin alone.

Platform	Principal Advantages	Typical Limitations	Most Credible Translational Role
Collagen/gelatin/GelMA	Biological familiarity; cell-interactive motifs; enzymatic remodelling; GelMA offers tunable photocrosslinking and printability [14].	Low intrinsic mechanical strength; crosslinking-dependent diffusion and degradation; batch/source variability.	Cell-instructive matrix, local delivery platform and printable bioink within a mechanically stabilised reconstruction.
Hyaluronic acid	Highly hydrated ECM-associated polymer; chemically modifiable; established use in injectable and clinically adjacent regenerative systems [18].	Rapid degradation unless modified; weak intrinsic mechanics; performance depends strongly on crosslink chemistry.	Injectable carrier, defect-conforming adjunct and component of composite graft or delivery systems.
Alginate	Mild ionic gelation; injectability; good encapsulation and delivery properties [19].	Limited intrinsic mammalian cell adhesion; mechanically compliant; ion-exchange-dependent stability.	Carrier for cells and biofactors, particularly when functionalised or reinforced.
Chitosan	Biodegradable cationic polymer; injectable formulations; potential antimicrobial contribution [21].	Formulation/pH sensitivity; modest mechanics; variable degradation and processing requirements.	Injectable composite for local anti-infective and regenerative use.
Silk fibroin and other protein systems	Favourable cytocompatibility and adjustable processing; can be functionalised for osteogenic signalling [17].	Processing-dependent structure and degradation; limited standalone load-bearing capacity.	Reinforced or functionalised matrices and bioinks.
Hybrid/mineralised composites	Combine biological polymer phase with osteoconduction, ion release or mechanical reinforcement [12,13].	Greater manufacturing, toxicology and regulatory complexity; risk of sacrificing injectability or degradability.	Most plausible route to defect-specific multifunctionality when complexity remains controllable.

**Table 2:** Translational maturity of representative natural-based hydrogel strategies. The table is intentionally ordered by evidence context rather than by material novelty.

Representative System/Indication	Highest Level of Evidence Represented Here	Key Finding	Translational Interpretation
rhFGF-2 in gelatin hydrogel—fresh tibial shaft fracture	Randomised double-blind placebo-controlled human trial	Accelerated radiographic union after intramedullary nailing; no significant difference in time to painless full-weight bearing [61].	Proof that a natural hydrogel can generate a measurable human long-bone effect; not evidence for established non-union or segmental-defect reconstruction.
Hyaluronan/BMP-2—standardised cranial defects	Prospective randomised human study	Substantial cranial-defect healing without treatment-related local or systemic adverse effects in the reported cohort [60].	Human regenerative evidence in a small non-load-bearing cranial setting.
Antibiotic-loaded DAC hydrogel—osteosynthesis/arthroplasty	Randomised clinical trials; 2026 network meta-analysis	Reduced early infection in reported trials without detectable impairment of fracture healing; broader pooled evidence remains heterogeneous [62,63,64].	Strong example of a bounded hydrogel function integrating into existing orthopaedic workflows; primarily anti-infective rather than regenerative.
BMP-2 CaP/hydrogel—segmental non-union	Canine critical-size segmental non-union model	Promoted bone regeneration in a large-animal defect [68].	More relevant mechanics and scale than calvarial models, but still preclinical.
FGF-2 gelatin hydrogel—fracture healing	Non-human primate fracture model	Accelerated healing and increased mechanical strength [69].	Illustrates a successful large-animal-to-human translational sequence.
Cell-laden/prevascularised constructs	Rodent craniofacial and femoral defect models	Enhanced bone formation, vascularisation or defect bridging [66,67].	Biologically strong proof of concept; manufacturing, scale and load remain unresolved.
Antimicrobial/immunomodulatory hydrogels—infected bone	Predominantly small-animal models	Local antibacterial action can be combined with preserved or enhanced repair [74,75].	Promising adjunct concept; clinical use must remain integrated with accepted FRI surgery and antimicrobial therapy.
EV/exosome-loaded hydrogels	Preclinical bone-defect models	Improved local retention, osteogenesis and angiogenesis [41,42].	High biological promise, but product identity, potency, scale-up and regulatory classification remain immature.

## Data Availability

No new data were created or analyzed in this study. Data sharing is not applicable to this article.
